# How Caregivers of People With Dementia Search for Dementia-Specific Information on the Internet: Survey Study

**DOI:** 10.2196/15480

**Published:** 2020-05-19

**Authors:** Areti Efthymiou, Evridiki Papastavrou, Nicos Middleton, Artemis Markatou, Paraskevi Sakka

**Affiliations:** 1 Department of Nursing Cyprus University of Technology Limassol Cyprus; 2 Consulting Management Training (CMT) Prooptiki Athens Greece; 3 Athens Association of Alzheimer's Disease and Related Disorders Athens Greece

**Keywords:** caregivers, internet, dementia, eHealth

## Abstract

**Background:**

During the last decade, more research has focused on web-based interventions delivered to support caregivers of people with dementia. However, little information is available in relation to internet use among caregivers in general, especially those caring for people with dementia.

**Objective:**

The aim of this study was to evaluate the dementia-related internet use and factors that may be associated with its use among caregivers of people with dementia in Greece.

**Methods:**

Secondary data from the Greek Dementia Survey of the Athens Association of Alzheimer’s Disease and Related Disorders were collected from April to June 2017. A total of 580 caregivers of people with dementia participated in the study.

**Results:**

The majority of the caregivers reported that they had used the internet in the previous 3 months (84.1%, 488/580). Nearly half of the caregivers (47.5%, 276/580) reported that they had received dementia services online. Bivariate analysis showed that a dementia-specific search of information was associated with age, education, kinship, and years of care. Age (odds ratio [OR] 2.362, 95% CI 1.05-5.33) and education (OR 2.228, 95% CI 1.01-4.94) were confirmed as predictors, with younger caregivers and those with higher educational attainment being more likely to search for dementia-specific information. Use of the internet to search for dementia information was only related to hours of care. The internet use by caregivers within the previous 3 months was associated with variables such as age, education, occupation, kinship, years of care, and self-reported impact on physical and social health.

**Conclusions:**

Caregivers of people with dementia in Greece, as in the other southern European countries, are essential agents of the national health system. The existing short- and long-term respite care services are limited or nonexistent. Currently, caregivers receive mostly support and education from memory clinics and municipality consultation centers, which are mainly based in central cities in Greece. Despite the dementia awareness movement in Greece, there is still space to integrate the role of technology in the support and education of caregivers. Development of training programs for enhancing electronic health literacy skills as well as web-based services provision could support Greek caregivers in their everyday caring tasks.

## Introduction

Caregivers of frail older people and people with dementia are a significant component of the national health care system across European countries [1]. Caring for a person with dementia is a demanding job, resulting in difficulty for caregivers to maintain their physical, psychological, and social health. Previous research indicates that caregivers are a more vulnerable group than the general population with problems ranging from social isolation; feelings of anger, guilt, grief, depression, and physical exhaustion; and difficulties in the reconciliation of work and care responsibilities [2-4].

The recent focus of research on web-based interventions supporting informal caregivers of people with dementia in their everyday tasks has revealed the role that these interventions will play in the coming years. Caregiver platforms providing psychoeducation, training, and other interventions, including telemedicine and other telehealth services such as mobile apps (eg, medication reminders, cognitive and reminiscence training, relaxation techniques, and forums), are only a few examples of the existing services that are accessed through the use of technology [5-9]. According to a recent scoping review [8], the majority of articles examining these interventions did not provide adequate information on use of the technology by caregivers after the end of the intervention. In the same review, the majority of the caregivers stated that the interventions were acceptable and usable. Therefore, there is a clear gap between the web-based interventions for caregivers and their overall web-based usage.

In the concept mapping presented by Chiu and Eysenbach [10], information and communication technology (ICT) factors, caregivers’ needs, and style of use are three core categories that influence the use of web-based interventions for caregivers of people with dementia. The accessibility, required effort, and style of use (interactive or passive) in combination with the social support, caregiving beliefs, self-efficacy, and years of caregiving reflect the complexity in understanding caregivers’ web-based service use. In this theoretical model, it is also relevant to add the component of electronic health (eHealth) literacy-related skills. Caregivers could benefit from the use of web-based services as they are cost-effective and easily accessible. However, this will only be successful if caregivers obtain or enhance their eHealth literacy skills. eHealth literacy is a concept that was initially defined by Norman and Skinner [11] in 2006, but the definition continues to trouble researchers [12].

Although studies have been performed to evaluate the factors influencing the health-related internet use of older people, little information is available on the specific population of family and other informal caregivers [13]. Research on the health-related internet use of older people confirms age and education as strong predictors of internet use, giving the advantage to younger and more educated people [14-18]. The number of electronic devices also seems to be a predictor of internet use [15]. Low income, low socioeconomic status, and racial/ethnic minorities are considered as predictors of internet nonuse [16]. Lack of skills when navigating the internet was the most common problem identified within a sample of people with rheumatic diseases, including difficulty in operating the computer and internet browser, navigating and orientating, utilizing search strategies, evaluating relevance and reliability, adding personal content to the web, and protecting and respecting privacy [19].

Age, gender, and occupation are associated with frequency of internet use among caregivers, even though they typically use the internet less frequently than the general public [13,20]. Caregivers seem to prefer to search for information on disease-specific websites and those related to patient organizations out of habit and accessibility to influence the search for information online [21]. Nevertheless, the doctor remains the primary source of information [21]. In a study of caregivers online (ALZconnected [22]), the authors identified the need for caregivers of people with dementia to post more emotion-related posts; queries about the disease did not exceed 12% of the posts.

According to Piirto et al [23], internet access in Greece increased by 28% between 2009 and 2014, with 49% of daily users in 2014 comprising the age group of 16 to 74 years. This percentage increased to 85% in 2017, according to updated data from Eurostat [24]. Typical activities include reading the news, finding information, email, social networking, health information search, downloading software, and searching for a job. Three in four people in the age group of 65 years and over reported using the internet daily [25]. In Greece, only 5% of people in the age group above 55 years searched the internet once a week for health-related information according to the Flash Eurobarometer 404 survey conducted in 2014. These searches usually included health promotion topics (eg, diet, exercise) and information on specific diseases and treatments [26].

The aim of the present study was to investigate the dementia-related internet use by caregivers of people with dementia in a sample of caregivers in Greece and to determine possible factors that may influence internet use, and consequentially the use of web-based interventions. This study is part of a more extensive project for the development and implementation of training services for caregivers of people with dementia. Our main aims were to determine (1) the information-seeking behavior among caregivers of people with dementia in Greece with a focus on internet use (dementia-specific information on the internet or other sources, and use of web-based services); (2) whether information-seeking behavior differs according to caregiver sociodemographic characteristics; and (3) the preferred training delivery method for caregivers of people with dementia in Greece.

## Methods

### Study Design

This study followed a descriptive study design to identify associations among caregiver characteristics and information-seeking behavior with a focus on internet use. The methodology followed the Checklist for Reporting Results of Internet E-Surveys [27]. Secondary data obtained from the Greek Dementia Survey of the Athens Association of Alzheimer’s Disease [28], a password-protected survey, were analyzed. The Greek Dementia Survey represents the first study among caregivers of people with dementia at a national level to investigate the educational needs, care service awareness, and style of internet use.

The survey was distributed by email or in hard copy form provided by the social workers of dementia centers. The aim of the Greek Dementia Survey included identifying demographics, training needs, available services for caregivers, and type of web-based services used. In total, the Greek Dementia Survey included 40 questions using multiple-choice, dichotomized responses (yes/no), or Likert-scale responses focusing on the caregivers’ training needs. The items were developed as part of a literature review and the consensus meetings of the experts in this area, including stakeholders such as the Athens Association of Alzheimer’s Disease and researchers focusing on dementia research. The electronic questionnaire was developed by the survey agency and was pretested within the research team (5 members). A survey agency organized the data collection from April to June 2017.

The data from 23 survey questions (including sociodemographic characteristics) were used for the present analysis (Textbox 1).

**Items included in the Greek Dementia Survey for the present analysis.**

- Sociodemographic characteristics: gender, education, age groups, occupation, financial status, caregiver relationship to patient, family status, type of caregiver, and period of caring
- Self-reported impact of caregiving (1 item)
- Search for dementia-specific information (6 items), training delivery preferences, and caregivers’ perceptions regarding the satisfaction derived from the information found
- Internet use for dementia-related information

1. “Do you or any close relative have internet access?”

2. “Have you used the internet in the previous 3 months?”

3. “Have you used a smartphone in the previous 3 months?”

4. “In the previous 6 months, have you received any dementia-related services (list) via the telephone, internet, face-to-face visit with an expert, or did not receive any such service.”

5. “If you have received any of the above services via the internet, please mention if it was accessed through a website, a social network, email, forum, video, blog FAQ, eLearning, teleconferencing, Quiz, or Chat.”

### Survey Administration

A total of 580 primary and secondary caregivers of people with dementia participated voluntarily in the survey by replying to online or to face-to-face questionnaires. No incentives were offered for the caregivers’ participation. The sample was identified from Athens Association of Alzheimer’s Disease registries and social media advertisement.

The survey was disseminated for 2 months through social media of the Athens Association of Alzheimer’s Disease, and health care professionals also informed dementia daycare center members of the survey. For those with access to email, the survey was emailed through a link. A unique access code was provided to every participant. The access code was stored together with the survey results to eliminate duplicate entries. The participants could save their responses and return to complete the survey, or they could edit or clear the replies and initiate the survey another time. The survey comprised a total of 6 screens, including the consent page. The duration of the survey ranged from 5 to 23 minutes, with a mean time stamp of 10 minutes. If the caregivers could not access the internet, health care professionals of the dementia centers of the Athens Association of Alzheimer’s Disease administered the survey as a face-to-face interview.

The inclusion criteria were as follows: caregivers over 18 years old, either primary or secondary (friend or family supporting the primary caregiver), of a person with dementia, helping the person in activities of daily living, and being capable of reading and writing in the Greek language. As there is limited research on internet use among caregivers of people with dementia, we expanded the inclusion criteria to both primary and secondary caregivers, since age is a factor influencing internet use and secondary caregivers are usually children younger than the primary caregiver. After the questionnaires were submitted, a completeness check was performed. In total, 31 of the 580 surveys (5.3%) were incomplete.

### Statistical Analysis

All data received by the email survey were entered manually into Statistical Package for the Social Sciences software (SPSS Inc, Chicago, IL, USA) by two researchers (NK and AM) who are staff members of the survey agency. Incomplete questionnaires were also analyzed. For analysis of the secondary data, descriptive statistics for caregiver characteristics and the replies received were computed. Bivariate analysis and binary logistic regression (backward conditional method) were performed.

### Ethics Approval and Informed Consent

Permission to conduct the study was granted by the Scientific Committee of the Athens Association of Alzheimer’s Disease, and was approved by the Executive Board on March 14, 2017.

Caregivers expressing interest in participating in the study were informed about the aim of the study, the length of time, and data storage by a researcher of the Athens Association of Alzheimer’s Disease (NK). At the end of the study, only the two data analysts (AM and AL), who are staff members of the survey agency, and the primary investigator (AE) had access to the data. Data will be stored for 5 years from the end of data collection. The researcher only used the email addresses of the caregivers to provide the link and the access code of the survey. The researcher (NK) invited the members of the Athens Association of Alzheimer’s Disease to participate, and after obtaining their consent, emailed them the survey link and access code. The participants that were not members of the association were informed about the survey by the association’s social media page and then emailed or messaged the researcher directly requesting the survey link.

## Results

Female caregivers and adult children caring for their parents constituted the majority of the sample. The majority of the participants considered themselves to be primary caregivers who undertook all of the responsibility or shared equal responsibility rather than as secondary caregivers. Caregivers under 65 years old were most highly represented among age groups. Nearly half of the caregivers were employed and about a quarter of the participants were pensioners. The majority of the caregivers had attained tertiary-level education and were married or cohabitating with a partner. Regarding the duration of care, the majority of caregivers had been caring for fewer than 5 years, with about a third of participants being caregivers for more than 5 years. Nearly half of the participants reported living with difficulty or needing to borrow money (Table 1).

Caring tasks had a negative impact on the psychological and social health of the caregivers for 67.6% (392/580) and 57.1% (331/580) of the participants, respectively. The majority of the caregivers (94.7%, 549/580) searched for dementia information in multiple ways (telephone, internet, printed material, available services, health professionals), and among those who replied positively, 54.6% (300/549) frequently searched for information and only 19.3% (106/549) reported that the information they found met their needs.

Nearly two in three (59.7%, 346/580) of the participants considered education about dementia to be very important. Nevertheless, only a small percentage of participants stated a preference to receive the training from eLearning (114/580, 19.7%) or a videoconferencing tool (34/580, 5.9%). Over a third of the participants (218/580, 37.6%) stated a preference for a blended learning training program with both face-to-face seminars and eLearning courses, with a similar proportion stating a preference for face-to-face training alone (207/580, 35.7%). The remaining participants indicated a preference for obtaining information from printed material (5/580, 0.8%) or other sources (0.5%).

Only a small percentage of the caregivers (5.3%, 31/580) did not access the internet. The majority of the caregivers reported that they had used the internet in the previous 3 months (84.1%, 488/580). They also reported that they used the internet mainly through smartphones or tablets (82.8%, 404/488) and almost half of the total sample (47.6%, 276/580) received dementia services online. Furthermore, the majority of caregivers reported that they had learned about available dementia services through the internet, followed by those informed by their doctor as a second source of information. The detailed breakdown of the sources of information is provided in Table 2.

Among the total sample, the majority of caregivers reported searching for online information about the disease (38.4%, 223/580), practical issues (23.3%, 135/580), available services (17.9%, 104/580), nonpharmacological interventions for people with dementia (10.7%, 62/580), and support and self-help advice for caregivers (7.8%, 45/580). They also reported that they do not frequently use the internet to interact with other caregivers or health care professionals to find out about financial issues, services related to patient safety, telemedicine, working caregiver support services, and mobility services for the person with dementia.

Caregivers who searched and used dementia services online within the previous 6 months mostly searched through websites (82.6%, 228/276), social media (28.3%, 78/276), and emails (21.4%, 59/276). The majority of caregivers searched in forums, blogs, or acquired information from videos, eLearning, teleconferences, quizzes, and chatrooms.

Regarding searching for dementia-specific information, we found statistically significant associations for women (χ_2_=18.000, *P*<.001), younger age (χ_2_=10.865, *P*=.03), higher education (χ_1_=8.288, *P*=.02), employed (χ_2_=14.126, *P*=.007), caring for a parent (χ_2_=7.994, *P*=.012), fewer hours of care (χ_2_=17.698, *P*<.001), and fewer than 5 years of care (χ_2_=18.000, *P*<.001) (Table 1).

Binary logistic regression analysis confirmed the associations of age and education. Caregivers under 65 years (odds ratio [OR] 2.362, 95% CI 1.05-5.33, *P*=.04) and those with more than 12 years of schooling (OR 2.228, 95% CI 1.01-4.94, *P*=.05) were more likely to search for dementia-specific information.

**Table 1:** Caregiver demographics and their association with dementia-specific information searching (N=580).

Characteristic		Respondents, n (%)		Dementia-specific information search							
					Frequently, n (%)		Occasionally, n (%)		Never, n (%)		*P* value
**Gender**										<.001	
	Women		430 (74.1)		244 (56.7)		166 (38.6)		20 (4.7)		
	Men		150 (25.9)		55 (36.7)		84 (56.0)		11 (7.3)		
**Age (years)**										.03	
	<65		483 (83.3)		254 (52.6)		209 (43.3)		20 (4.1)		
	66-85		93 (16 .0)		44 (47)		38 (41)		11 (12)		
	>86		4 (0.7)		1 (25)		3 (75)		0 (0)		
**Education**										.02	
	<12 years of secondary education		237 (40.9)		123 (51.9)		94 (39.7)		20 (8.4)		
	Tertiary education		343 (59.1)		176 (51.3)		156 (45.5)		11 (3.2)		
**Occupation**										.007	
	Unemployed/student/homemaker		140 (24.1)		83 (59.3)		48 (34.3)		9 (6.4)		
	Employed		299 (51.6)		141 (47.2)		148 (49.5)		10 (3.3)		
	Pensioner		141 (24.3)		75 (53.2)		54 (38.3)		12 (8.5)		
**Financial status^a^**										.48	
	Living comfortably		137 (23.6)		65 (94.9)		65 (47.4)		7 (5.1)		
	Living with no major difficulties		174 (30)		85 (48.9)		82 (47.1)		7 (4.0)		
	Living with difficulty/borrow money		259 (44.7)		144 (55.6)		99 (38.2)		16 (6.2)		
**Family status**										.85	
	Married/cohabitation		379 (65.4)		201 (53.0)		157 (41.4)		21 (5.5)		
	Single/divorced		186 (32.1)		91 (48.9)		86 (46.2)		9 (4.8)		
	Widowed		15 (2.6)		7 (47)		7 (47)		1 (7)		
**Caregiver relationship to patient**										.19	
	Child		389 (67.1)		202 (51.9)		171 (44)		16 (4.1)		
	Spouse		112 (19.3)		62 (55.4)		41 (36.6)		9 (29)		
	Other		79 (13.6)		35 (44)		38 (48)		6 (8)		
**Type of caregiver**										.02	
	Primary caregiver or sharing equally		396 (68.3)		215 (54.3)		156 (39.4)		25 (6.3)		
	Secondary caregiver		184 (31.7)		84 (45.7)		94 (51.1)		6 (3.3)		
**Hours of care per week^b^**										<.001	
	<20		301 (51.9)		136 (45.2)		154 (51.2)		11 (3.7)		
	>20		279 (48.1)		163 (58.4)		96 (34.4)		20 (7.2)		
**Period of caregiving (years)**										.005	
	<5		389 (67.1)		183 (47.0)		181 (46.5)		25 (6.4)		
	>5		191 (32.9)		116 (60.7)		69 (36.1)		6 (3.1)		

^a^Ten caregivers did not provide responses related to financial status.

^b^Mean 38.59, SD 45.92 (median 20).

**Table 2:** Source of information about available dementia services (N=388^a^).

Source	n (%)
Internet	154 (39.7)
Doctor caring for the person with dementia	92 (23.7)
Event/seminar	76 (19.6)
Friend/acquaintance	74 (19.1)
Informational material, journal, or newsletter	71 (18.3)
Television	49 (12.6)
Family	36 (9.3)
Health care professional in dementia care	24 (6.2)
Radio	12 (3.1)
Paid caregiver	2 (0.3)
Other	6 (1.5)

^a^This table presents the responses of 388 caregivers who replied that they knew about the available dementia services.

Internet use by caregivers was associated with younger age (χ_1_=141.27, *P*<.001), higher education (χ_1_=46.23, *P*<.001), employed (χ_1_=49.273, *P*<.001), caring for a parent (χ_2_=111.61, *P*<.001), being married (χ_1_=8.574, *P*=.01), caring for fewer than 5 years (χ_1_=6.70, *P*=.01), caring for fewer than 20 hours per week (χ_1_=12.83, *P*<.001), and reporting a physical impact (χ_1_=7.76, *P*=.005) and a social health impact (χ_1_=4.76, *P*=.03). According to the binary logistic regression with the backward conditional method, age, education, kinship, and caring period were confirmed as significant predictors of internet use. Caregivers in the age group under 65 years were almost 5 times more likely to use the internet in comparison with those of the age group 66-85 years. Caregivers with higher education (>12 years) and being children of the patient were 3 times more likely to use the internet, and caregivers with fewer than 5 years of caring were almost 2 times more likely to use the internet (Table 3).

Searching for dementia-related information online was only significantly associated with the hours of care (χ_1_=10.461, *P*=.005).

**Table 3:** Predictors of internet use based on binary logistic regression.

Independent variable	Odds ratio	95% CI	*P* value
Age (reference category: 66-85 years)	5.096	2.324-11.177	<.001
Education (reference category: <12 years)	2.940	1.669-5.178	<.001
Kinship (1) (reference category: spouse)	3.257	1.395-7.603	.006
Kinship (2) (reference category: other relatives)	3.387	1.523-7.531	.003
Caring period (reference category: >5 years)	1.788	1.027-3.112	.04

## Discussion

### Principal Findings

The present study aimed to identify the dementia-specific information-searching behaviors among caregivers of people with dementia in Greece using the internet or other sources, as well as their preferences regarding the web-based tools and modes of dementia training delivery. This topic is quite innovative for this specific population, as there is no relevant research available in Greece.

The Eurostat internet use report [24] indicated that Greece had one of the lowest percentages of internet use among people 16 to 75 years old in 2018. Thus, reporting the caregivers’ search behavior is a first step to recognize this issue among caregivers and to raise awareness regarding the specific health and eHealth literacy skills that are important for adapting to the new technological era. Age and education of this sample were associated with searches for dementia-specific information.

Age and education are two variables that are strongly related to health literacy levels, and the question related to the use of a dementia-specific information search could be considered as a screening question for this population regarding their health literacy. For caregivers to search for dementia-specific information, they require the necessary motivation, knowledge, and skills, which are the three core elements according to Soerensen et al [29] in the health literacy model.

Kim [13] examined the prevalence and searched for factors related to health-related internet use among caregivers of people with dementia based on responses to the question: “How often, if at all, have you gone on internet websites in the past year to find information and resources in any way related to being a caregiver for your care recipient?” Health-related internet use was associated with younger age, higher education, fewer hours per week in caring tasks, emotional stress, and financial difficulties in comparison with nonhealth-related internet use. In the present study, searching for dementia-specific information on the internet was associated with hours of care; however, the percentage of caregivers searching for dementia-specific information on the internet was slightly lower than that reported by Kim (47.5%). In addition, internet use by caregivers was associated with age, education, kinship, and years of care. Caregivers participating in this survey responded that they did not use the internet as a source of interaction with other caregivers or health care professionals, and forums, chatrooms, blogs, eLearning, and videoconferencing were the least used services among caregivers. They also preferred a blended training approach with the use of face-to-face meetings and eLearning.

In Greece, apps tailored to caregivers’ needs have only been developed in the last 4 years as part of European research projects, and with the collaboration of Alzheimer disease associations [9]. Disease-specific associations usually develop their own informational websites. An informative platform for caregivers of older people was also developed as part of a European-funded project [6]. Social media groups for caregivers were only created in the last 2 years in Greece. However, according to our results, caregivers do not frequently use these types of services. In a related study, networking with other caregivers, facilitating interactions, and developing technologies that reflect daily experiences were reported as essential needs of the internet use by caregivers and were considered to be more critical than searching for information on care provision [30]. Therefore, our finding may reflect the lack of skills for use of the specific services by this population in combination with the lack of web services in Greece tailored to the needs of caregivers. According to Chiu and Eysenbach [10], the accessibility, perceived effort required, social support, personal skills, and beliefs, as well as the years of caregiving and the way in which a person uses the internet are all factors that influence the pattern of ICT intervention use by caregivers. Therefore, eHealth literacy skills should also be added as part of personal skills. In addition, a fourth higher-order category needs to be added together with the ICT factors reflecting caregiving needs and style of use. This category would include perspectives of health care professionals of ICT use, who should be involved in the development, implementation, and dissemination of caregiver-specific ICT tools.

In Greece, as in other southeastern European countries, caregivers of people with dementia are essential agents of the national health system. The existing short- and long-term respite care services are limited if nonexistent. Currently, caregivers receive mostly support and education as part of the services of memory clinics and municipality consultation centers, which are usually based in the central cities in Greece. Despite the dementia awareness movement in Greece, there is still space to integrate the role of technology in the support and education of caregivers. In 2016, the first eHealth literacy study was performed among Greek citizens reporting the eHealth literacy levels among different age groups and concluding the importance of age and education as predicting factors [31]. Research regarding the eHealth literacy level among caregivers of older people in Greece and Cyprus was only published for the first time in 2019 [32]. The caregivers in Greece and Cyprus reported a sufficient level of eHealth literacy skills (eHeals-Carer total score 29.70, SD 5.30, range 8-40) in comparison with available data [33-35]. We consider that the role of technology will become of great assistance among caregivers, as it will facilitate their everyday tasks, and may help to decrease the burden on the national health system. This could be achieved if caregivers enhance their skills to search for information and learn to evaluate and apply for them, not only from the internet but also from other sources. Nonprofit organizations and the existing dementia strategy could integrate training programs regarding the enhancement of health and eHealth literacy skills of caregivers of people with dementia in Greece, as the role of new technologies will become an integral part of our society in the coming years.

### Limitations and Strengths

This study included a set of questions on searching for dementia-specific information either on the internet or from other sources as part of the Greek Dementia Survey and was distributed mainly through the online registries of the Athens Association for Alzheimer’s Disease. Only a small number of participants answered the questions in hard copy form owing to difficulties in accessing or using the internet. Therefore, the low eHealth literacy caregivers were not adequately represented in the sample, who might provide clearer understanding of their difficulties in using everyday technology such as smartphones and apps.

Future studies could use a more heterogeneous sample with low or no knowledge of the internet to identify the needs of technology use in Greece. Despite this limitation, this is the first study among caregivers of people with dementia in Greece, which provides new knowledge of the internet use behavior and dementia-specific information-seeking online behavior for this population, laying the foundation for future research in this respect.

In Greece, as in other southeastern European countries, older people are not well accustomed to everyday technology. There are currently no available data of the internet use by people in Greece over 75 years old [36]. Children of people with dementia typically search for dementia-specific information to assist the primary caregivers. Primary caregivers, due to older age (in the case of spouses), may not know how to use the internet or are aware only of basic internet sources (eg, visiting specific websites to read the news or to play cognitive games). These factors could justify why the majority of the present sample included children caring for their parents.

### Conclusions

Internet use through tablets and smartphones has become part of everyday life in the last 20 years. Nevertheless, there is variation in internet use according to gender, age, education, socioeconomic, and cultural factors. In the next few years, training programs will be developed to enhance the informal learning of digital skills among older adults mainly as part of European projects or national digital strategies. The population above 65 years old remains a broad age group that has fewer opportunities in comparison with younger adults. This situation was partially confirmed by our study, since the age group that used the internet and searched for dementia information was mainly under 65 years old. Based on this finding, future interventions could implement the following three aspects: (1) nonprofit associations and vocational training organizations to provide tools to enhance the health and eHealth literacy skills of caregivers of people with dementia who are over 65 years old; (2) develop low-cost, easy to use ICTs tailored to the specific needs of caregivers; and (3) raise awareness of ICT for caregivers among health care professionals. Future research should also focus on measuring the level of eHealth literacy among caregivers of people with dementia in Greece, identifying the specific technological needs in everyday life, and piloting training programs integrating the enhancement of health and eHealth literacy skills among caregivers and health care professionals.

## Abbreviations

eHealth: electronic health
ICT: Information and communication technologies
OR: odds ratio
